# Serious adverse events following treatment of visceral leishmaniasis: A systematic review and meta-analysis

**DOI:** 10.1371/journal.pntd.0009302

**Published:** 2021-03-29

**Authors:** Sauman Singh-Phulgenda, Prabin Dahal, Roland Ngu, Brittany J. Maguire, Alice Hawryszkiewycz, Sumayyah Rashan, Matthew Brack, Christine M. Halleux, Fabiana Alves, Kasia Stepniewska, Piero L Olliaro, Philippe J. Guerin

**Affiliations:** 1 Infectious Diseases Data Observatory – IDDO, Oxford, United Kingdom; 2 Centre for Tropical Medicine and Global Health, Nuffield Department of Medicine, University of Oxford, Oxford, United Kingdom; 3 UNICEF/UNDP/WB/WHO Special Program for Research and Training in Tropical Diseases (TDR), World Health Organization, Geneva, Switzerland; 4 Drugs for Neglected Diseases initiative, Geneva, Switzerland; Universidade Federal de Minas Gerais, BRAZIL

## Abstract

**Background:**

Despite a historical association with poor tolerability, a comprehensive review on safety of antileishmanial chemotherapies is lacking. We carried out an update of a previous systematic review of all published clinical trials in visceral leishmaniasis (VL) from 1980 to 2019 to document any reported serious adverse events (SAEs).

**Methods:**

For this updated systematic review, we searched the following databases from 1^st^ Jan 2016 through 2^nd^ of May 2019: PUBMED, Embase, Scopus, Web of Science, Cochrane, clinicaltrials.gov, WHO ICTRP, and the Global Index Medicus. We included randomised and non-randomised interventional studies aimed at assessing therapeutic efficacy and extracted the number of SAEs reported within the first 30 days of treatment initiation. The incidence rate of death (IRD) from individual treatment arms were combined in a meta-analysis using random effects Poisson regression.

**Results:**

We identified 157 published studies enrolling 35,376 patients in 347 treatment arms. Pentavalent antimony was administered in 74 (21.3%), multiple-dose liposomal amphotericin B (L-AmB) in 52 (15.0%), amphotericin b deoxycholate in 51 (14.7%), miltefosine in 33 (9.5%), amphotericin b fat/lipid/colloid/cholesterol in 31 (8.9%), and single-dose L-AmB in 17 (4.9%) arms. There was a total of 804 SAEs reported of which 793 (including 428 deaths) were extracted at study arm level (11 SAEs were reported at study level only). During the first 30 days, there were 285 (66.6%) deaths with the overall IRD estimated at 0.068 [95% confidence interval (CI): 0.041–0.114; I^2^ = 81.4%; 95% prediction interval (PI): 0.001–2.779] per 1,000 person-days at risk; the rate was 0.628 [95% CI: 0.368–1.021; I^2^ = 82.5%] in Eastern Africa, and 0.041 [95% CI: 0.021–0.081; I^2^ = 68.1%] in the Indian Subcontinent. In 21 study arms which clearly indicated allowing the inclusion of patients with HIV co-infections the IRD was 0.575 [95% CI: 0.244–1.355; I^2^ = 91.9%] compared to 0.043 [95% CI: 0.020–0.090; I^2^ = 62.5%] in 160 arms which excluded HIV co-infections.

**Conclusion:**

Mortality within the first 30 days of VL treatment initiation was a rarely reported event in clinical trials with an overall estimated rate of 0.068 deaths per 1,000 person-days at risk, though it varied across regions and patient populations. These estimates may serve as a benchmark for future trials against which mortality data from prospective and pharmacovigilance studies can be compared. The methodological limitations exposed by our review support the need to assemble individual patient data (IPD) to conduct robust IPD meta-analyses and generate stronger evidence from existing trials to support treatment guidelines and guide future research.

## Author summary

Visceral leishmaniasis, also known as Kala-Azar, is a neglected infectious disease of poverty affecting countries in the Indian Subcontinent and Eastern Africa. Existing treatments have concerning safety profiles and are far from ideal, with documented reports of serious adverse events (SAEs) following therapy. We did a systematic review of all published clinical trials in VL (1980–2019) to document SAEs following treatment administration reported in clinical literature and carried out a meta-analysis to quantify the incidence rate of mortality within 30 days of treatment initiation. We found that safety outcomes are poorly reported in published literature with substantial missing information regarding timing and frequency of the events. We also observed that mortality following treatment with antileishmanial drugs, as reported in the literature, is a rare event. There was substantial variability in mortality rates across geographical regions and patient sub-groups. Results from this review can provide benchmark estimates for comparing data from prospective trials and pharmacovigilance studies and support the need to assemble individual patient data (IPD) to conduct a robust IPD meta-analyses to overcome some of the underlying limitations.

## Introduction

Visceral Leishmaniasis (VL) is a vector-borne disease caused by protozoan parasites of the genus *Leishmania*. The disease is endemic in parts of South Asia, East Africa, South America and the Mediterranean region with an estimated 50,000 to 90,000 cases occurring annually [1]. VL is mainly caused by *L*. *donovani* in East Africa and the Indian Subcontinent (ISC), and by *L*. *infantum* in the Mediterranean region and South America [2]. VL continues to exert substantial burden and is a leading cause of morbidity in the regions where the disease is endemic [3]. Untreated symptomatic forms of VL are almost always fatal, with the mortality dropping to approximately 5% among those who are treated [4].

The safety of antileishmanials remains a distinct cause of concern despite their success in achieving a clinical cure, especially when deployed at scale in routine control programmes. The poor tolerability of VL therapeutic options was established as early as the first decades of the 20^th^ century. Empiric treatment with tartar emetic, a trivalent antimony, produced toxicity that outweighed their therapeutic benefit to the patients in several cases [5]. Dose-dependent toxicities including prolonged QT interval, cardiac arrhythmia [6,7] and *Torsade de Pointes* [8] have been reported following pentavalent antimony (PA), the successor of trivalent antimony. Despite these known toxicities, absence of alternatives meant that PA remained the mainstay treatment against VL for much of the 20^th^ century. Several therapies were tested but safety continued to be a major hurdle in drug development: pentamidine which was introduced in the 1950s was associated with triggering insulin-dependent diabetes mellitus [9]; sitamaquine was associated with glomerulonephritis, which caused its development to be terminated [10,11]; unacceptable toxicity precluded the use of amphotericin B deoxycholate among patients with renal impairment and confined its usage mainly to teaching and district hospitals in India [12]. The discovery that encapsulation of deoxycholate into a liposomal membrane (liposomal amphotericin B) allows a lower drug exposure in the plasma leading to improved safety heralded a new era in the treatment of VL [13].

The liposomal form of amphotericin B has since been explored in several trials in Asia, Africa and Latin America and is well tolerated [4]. It has been tested in three different formulations: colloidal dispersion amphotericin B, lipid complex amphotericin B, and liposomal amphotericin B (L-AmB). The latter is currently deployed either as a single-dose regimen (10 mg/kg) [14] or as multiple doses administered over a treatment period often lasting 3–10 days (with a total dose up to 40 mg/kg) [2,15]. The practical advantages offered by the single-dose L-AmB regimen (10 mg/kg), coupled with high efficacy and acceptable safety profile in clinical trial settings, has led it to become the regimen of choice as the frontline drug in the ongoing Kala-Azar elimination programme (KAEP) in Bangladesh, India, and Nepal [16]. The KAEP requires that patients are followed-up for at least six months post-treatment to gauge events like relapse, death or any other serious adverse events (SAEs) (serious adverse events are defined as any undesirable experience associated with the study drug that leads to the following patient outcomes: life-threatening, prolonged hospitalisation, death, disability/permanent damage, or congenital/birth defect), and up to 3 years of follow-up is recommended to capture the incidence of post-kala-azar dermal leishmaniasis (PKDL) [17]. In practice, due to resource constraints, only few cases are followed-up. In addition to the study follow-up, the intrinsic inter- and intra-regional variation in drug efficacy [18], co-infection with HIV [4], and regional variation in age-sex distribution [19,20] might impact the safety and tolerability profile of the drugs. For example, among patients with HIV co-infections, Liposomal amphotericin B either as monotherapy or in a combination with miltefosine generally have been associated with favourable outcomes [2,15,21,22]. While the safety of antileishmanial is well-studied in clinical settings, only a handful of studies have characterised the tolerability profile of the drug when used in non-clinical trial settings [23,24]. If the expected event rates after drug administration in a controlled clinical trial setting are known, this might provide a baseline measurement for a more targeted surveillance in community settings and may serve as a benchmark for future trials against which mortality data from prospective and pharmacovigilance studies can be compared.

This systematic review of published clinical trials was conducted with an overarching aim of documenting reported serious adverse events (SAEs) and quantifying the observed mortality rate following drug administration in patients with visceral leishmaniasis.

## Material and methods

### Eligibility criteria

A systematic review of the existing scientific literature to identify clinical trials of therapies for VL was conducted in accordance with the Preferred Reporting Items for Systematic-Reviews and Meta-Analyses (PRISMA) guidelines (S1 Text) [25]. A pre-determined inclusion and exclusion criteria, described elsewhere, were used for study screening [26]. Briefly, all clinical trials in humans assessing the efficacy and safety outcomes of antileishmanial drugs as intervention were eligible for inclusion in this review. The review was not limited by comparators or language given the desire to capture all trials evaluating any treatment modality. Studies on PKDL, cutaneous leishmaniasis, canine visceral leishmaniasis, and those evaluating vaccines or prophylactic usage of drugs, vector control, net distributions, prevalence estimation and diagnostic tests were excluded.

### Information sources and search strategy

The identification of studies included in this study is the result of two separate literature searches. This review builds on a previous systematic review which had identified clinical trials registered or published before January 2016 [26]. The current search was designed to capture any VL clinical trial records published or registered between 1^st^ January 2016 and 2^nd^ of May 2019 (search was run on 2^nd^ of May 2019) in the following databases: PubMed, Ovid Embase, Scopus, Web of Science, Cochrane, clinicaltrials.gov, WHO ICTRP, and the Global Index Medicus. The update search was run by an experienced librarian at the Bodleian Health Care Libraries, the University of Oxford. The systematic review considered both randomised and non-randomised interventional studies including single-armed trials for the assessment of the serious adverse events (SAEs) [27]. Details of the search strategy adopted for each of the databases are provided in supplemental file (S2 Text).

### Study selection and data extraction

Two reviewers (MB, SR) screened the studies identified in the search in a blinded fashion using Covidence software for systematic reviews [28]. Any disagreement was resolved by contacting a third reviewer (BJM). Variable and data dictionaries with extraction rules were developed for the construction of a REDCap electronic data capture tool hosted at the University of Oxford [29].

Data on the following aspects were extracted from studies meeting inclusion criteria: study design, location, follow-up duration, the number of participants enrolled, information on study arms, and drug posology. Data on the following safety parameters were extracted: reports of hypersensitivity/allergic reaction to treatment regimen at dose testing, reports of SAEs and death during treatment or study follow-up.

The REDCap database was piloted with an initial extraction of 10 studies by two authors (AH and SSP) with the aim to simplify and standardise extraction rules. Two authors (RN and SSP) independently validated/updated the variables already extracted in the previous review [26] and extracted data from all the articles included in this review (each author extracted data from half of the studies). Any discordances that developed in the course of the data extraction were resolved through discussions for clarity and consistencies of the data being extracted among the two reviewers. In case of discrepancies, a resolution was reached with further discussions with two authors (PD, BJM). A detailed list of variables and metadata is provided in supplemental file (S1 Data).

### Statistical analyses and synthesis of results

#### Monitoring of adverse events in included studies

If there were any reports of AE (including death) during the treatment or the study follow-up; then the study was defined to have adverse events monitoring system (AEMS) in place. The standards used for coding/grading the AEs were extracted (when reported). If neither AEs were reported nor the standards were mentioned, the study was assigned “Unclear” AEMS status.

#### Hypersensitivity to the study drug

Data were extracted on dose-testing prior to the administration of amphotericin B formulations. The rate of allergic reactions leading to treatment discontinuation after dose testing was calculated using a random effects meta-analysis of single proportion using logistic transformation.

#### Serious adverse events (SAEs)

Data on SAEs were extracted as reported in the original publications. Due to difficulties in accurately extracting the unique number of patients who experienced SAEs at any time during the study treatment and follow-up period, the analysis of SAEs was limited to reporting descriptive statistics.

#### Estimation of rate of death

When the time of death was not explicitly stated, description of death “during the therapy or treatment period” was considered as death occurring within the first month (≤ 30 days) of treatment initiation, and deaths after discharge or during follow-up were considered as occurring after day 30. Similarly, in studies not reporting any information on death, it was assumed that no deaths had occurred. The incidence rate of death (IRD) within the first 30 days of treatment initiation from individual treatment arms were combined using random effects Poisson regression and were expressed as the number of deaths per 1,000 person-days at risk [30]. Heterogeneity was assessed using I^2^ statistics, which quantifies the proportion of total variability that is due to between-study differences [31]. The pooled estimates were presented together with the associated 95% confidence intervals (95% CIs). In addition, 95% prediction intervals (95% PIs) were also presented, which provides a range of values in which a future estimate of 30-day mortality rate in a set of VL patients will fall with probability of 0.95 [32]. The pooled incidence rates were adjusted using Copas selection model to account for potential publication biases [33,34]. Compared to other methods for testing publication bias (e.g., asymmetry of funnel plot), the Copas selection model explicitly models the publication bias by estimating the probability that a study is selected for publication conditional to the precision of the study result [33,34]. Mean difference in incidence rates were presented between sub-groups of interest and associated 95% confidence interval for the mean difference was constructed using delta rule (S3 Text). We further stratified the meta-analysis by whether the study allowed for inclusion of patients with HIV co-infections and geographical region.

#### Assessment of risk of bias

The assessment of the risk of bias in studies included in this review was carried out using the Cochrane Risk of Bias (ROB) tool for randomised controlled trials. Two reviewers independently assessed the risk of bias in randomised studies based on their design and conduct. Risk of bias in non-randomised studies were assessed using ROBINS-I tool (with modifications adopted for the current review) [35].

#### Sensitivity analyses

Sensitivity analysis was carried out by considering all deaths with unclear time of occurrence as occurring within the first 30 days of treatment initiation (worst case analysis). Estimated rates were also estimated separately for randomised and other than randomised studies (single-armed trials, non-randomised studies, alternative or consecutive treatment allocation or randomisation status not specified), and by the adjudicated risk of bias status on different domains.

#### Software

All statistical analyses were carried out using R software [36]. Meta-analysis of incidence rates was carried out using the metarate function in **meta** package and sub-group differences were explored using the test of moderator effects. Estimates adjusted for selection biases (using Copas selection model) were derived using the **metasens** package.

## Results

### Study selection and characteristics

**Fig 1:**
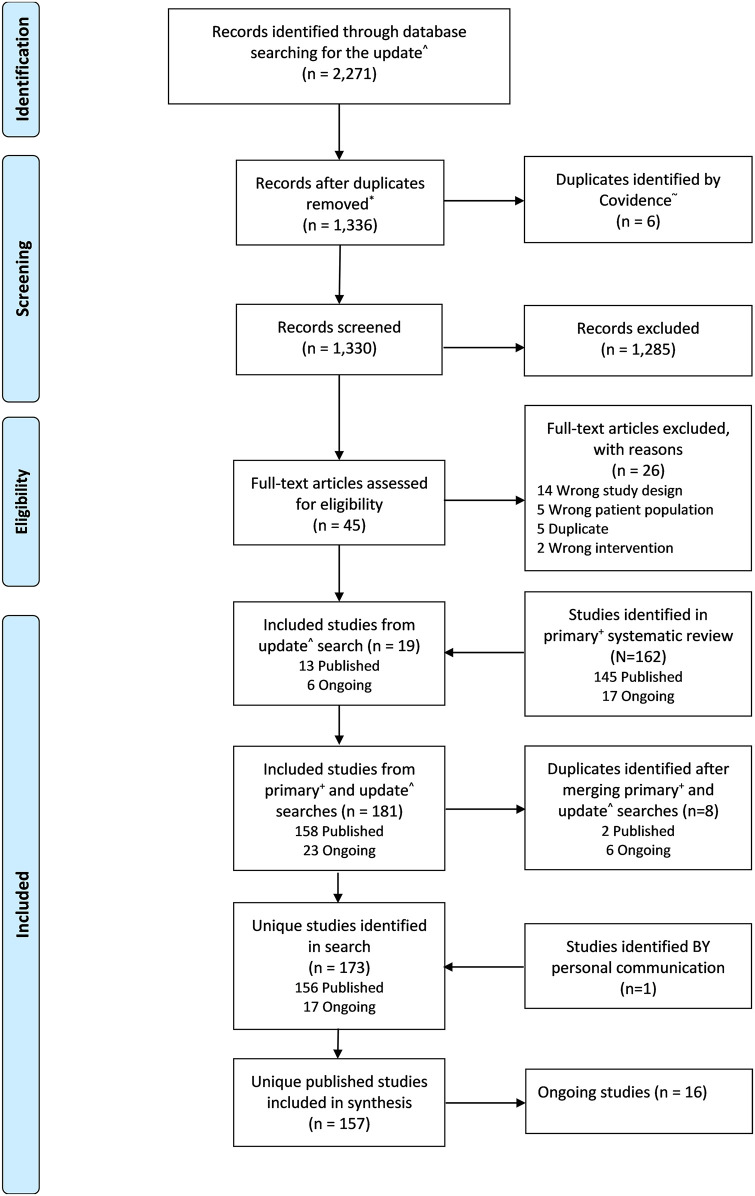
Preferred Reporting Items for Systematic Reviews and Meta-Analyses (PRISMA) flow diagram of publications screened. ^ Refers to the update literature search described in this manuscript, January 2016–02 May 2019. *De-duplicated by librarian. + Bush JT, Wasunna M, Alves F, Alvar J, Olliaro PL, Otieno M, et al. (2017) Systematic review of clinical trials assessing the therapeutic efficacy of visceral leishmaniasis treatments: A first step to assess the feasibility of establishing an individual patient data sharing platform. PLoS Negl Trop Dis 11(9): e0005781. ~ Covidence systematic review software, Veritas Health Innovation, Melbourne, Australia. Available at www.covidence.org.

There were 2,271 records identified from the updated literature searches for the period 1^st^ January 2016 to 2^nd^ of May 2019, of which 1,330 were unique. Of the 1,330 unique records, 1,285 were excluded at the level of title and abstract screening. Full texts of the remaining 45 records were assessed, of which only 19 met the eligibility criteria. All 162 studies identified in the previous systematic review (period 1980 to 2015) [26], met the eligibility criteria and were merged with the 19 newly identified studies. The merger identified 173 (156 published; 17 ongoing) unique studies after the removal of 8 duplicates. One ongoing study was published and identified through personal communication leading to a total of 157 studies published from 1980 through to 2019 included in this review for data extraction (Fig 1).

There were 105 (66.9%) studies from the Indian Subcontinent, 27 (17.2%) from Eastern Africa, 9 (5.7%) from the Mediterranean region, 7 (4.5%) from Southern America, 5 (3.2%) from Central Asia (Middle East), and 4 (2.5%) were multi-regional. A total of 64 (40.8%) studies were randomised, 54 (34.4%) were single-armed studies, 26 (16.6%) were non-randomised, 1 (0.6%) was partially randomised (multi-centre study with randomised allocation in only one of the study sites) [37], and the randomisation status was not described in 12 (7.6%). Patients with HIV positive status were enrolled in 15 (9.6%) studies, excluded in 73 (46.5%) studies, and their inclusion was unclear in 69 (43.9%) studies. The follow-up duration was less than 60 days in 7 (4.5%) studies, 180 days in 111 (70.7%) studies, between 181 to 365 days in 32 (20.4%) studies, 1020 days in 1 study (0.6%), and was not clear in 6 (3.8%) studies. Further details of the studies included are presented in supplemental file (S1 Data).

### Description of drug arms and patients treated

There was a total of 347 treatment arms of which 74 (21.3%) tested pentavalent antimony, 52 (15.0%) tested multiple-dose L-AmB regimen, amphotericin b deoxycholate was administered in 51 (14.7%) arms, miltefosine in 33 (9.5%) arms, amphotericin b fat/lipid/colloid/cholesterol in 31 (8.9%) arms, PA was tested in combination with other drug regimens in 20 (5.8%) arms, and single-dose L-AmB was tested in 17 (4.9%) arms. A total of 35,376 patients were enrolled of whom 26,441 (74.7%) were from the Indian Subcontinent (ISC), 7,361 (20.8%) were from Eastern Africa (Table 1). Amphotericin B monotherapies were tested in 12,651 patients of whom 11,278 (89.1%) were from the ISC, while PA was tested in 7,118 patients, of whom 4,384 (61.6%) were from the ISC and 2,210 (31.0%) were the EA region (Table 1).

**Table 1:** Drug regimens tested and number of patients treated for each regimen in the studies included in the review.

	Indian Subcontinent	Central Asia	Eastern Africa	Mediterranean	Southern America	Multi-Regional
**Pentavalent Antimony** (74 arms)	4,384 (16.6%)	211 (96.8%)	2,210 (30.0%)	115 (23.7%)	175 (26.8%)	23 (10.6%)
**Amphotericin B formulations**						
Amphotericin B deoxycholate (51 arms)	6,048 (22.9%)	-	210 (2.9%)	45 (9.3%)	148 (22.7%)	-
Amphotericin b fat/lipid/colloid/cholesterol (31 arms)	1,187 (4.5%)	-	-	50 (10.3%)	30 (4.6%)	6 (2.8%)
L-AmB (52 arms)	812 (3.1%)	-	195 (2.6%)	276 (56.8%)	166 (25.4%)	186 (85.7%)
L-AmB (Single dose) (17 arms)	3,231 (12.2%)	-	61 (0.8%)	-	-	-
**Combination Therapy with L-AmB**						
L-AmB (Single) + Miltefosine (7 arms)	816 (3.1%)	-	-	-	-	-
L-AmB (Single) + PA (1 arm)	-	-	-	-	112 (17.2%)	-
L-AmB (Single) + Paromomycin (1 arm)	159 (0.6%)	-	-	-	-	-
L-AmB + Miltefosine (3 arms)	160 (0.6%)	-	88 (1.2%)	-	-	-
L-AmB + PA (1 arm)	-	-	51 (0.7%)	-	-	-
L-AmB + Paromomycin (1 arm)	158 (0.6%)	-	-	-	-	-
**Miltefosine** (33 arm)	5,324 (20.1%)	-	371 (5.0%)	-	-	-
**Sitamaquine** (17 arm)	161 (0.6%)	-	113 (1.5%)	-	22 (3.4%)	-
**Paromomycin** (15 arm)	1,625 (6.1%)	-	382 (5.2%)	-	-	-
**Pentamidine** (9 arm)	707 (2.7%)	-	-	-	-	-
**Other combination therapies**						
**Paromomycin + Miltefosine** (3 arm)	813 (3.1%)	-	-	-	-	-
**Paromomycin + PA** (11 arm)	236 (0.9%)	-	3,608 (49.0%)	-	-	-
**Other regimens**					-	-
Ketoconazole (2 arms)	90 (0.3%)	-	7 (0.1%)	-	-	-
PA + Allopurinol (2 arms)	32 (0.1%)	-	23 (0.3%)	-	-	-
PA + Pentamidine (2 arms)	208 (0.8%)	-	-	-	-	-
Allulopurinol (1 arm)	-	7 (3.2%)	-	-	-	-
Aminosidine sulphate or Paromomycin (1 arm)	-	-	19 (0.3%)	-	-	-
Aminosidine sulphate or Paromomycin + SSG (1 arm)	-	-	23 (0.3%)	-	-	-
Aminosidine/paromomycin + SSG (1 arm)	24 (0.1%)	-	-	-	-	-
Atovaquone (1 arm)	15 (0.1%)	-	-	-	-	-
Atovaquone + Fluconazole (1 arm)	13 (0%)	-	-	-	-	-
Fluconazole (1 arm)	20 (0.1%)	-	-	-	-	-
Metronidazole (1 arm)	10 (0%)	-	-	-	-	-
PA + Ketoconazole (1 arm)	32 (0.1%)	-	-	-	-	-
PA + Levamisole (1 arm)	32 (0.1%)	-	-	-	-	-
PA + Verapamil (1 arm)	10 (0%)	-	-	-	-	-
Pentamidine + Allopurinol (1 arm)	80 (0.3%)	-	-	-	-	-
Roxithromycin (1 arm)	54 (0.2%)	-	-	-	-	-
No drug (1 arm)	-	-	-	-	-	2 (0.9%)
Column total	26,441	218	7,361	486	653	217

Column percentage shown in parenthesis; AMBd = amphotericin B deoxycholate; L-AmB = Liposomal amphotericin B; PA = pentavalent antimony; SSG = sodium stibogluconate

### Adverse events monitoring system

An adverse event monitoring system (AEMS) was reported to be in place for 149 (94.9%) studies whereas it was not clear for the remaining 8 (5.1%) studies. Of the 149 studies that reported AEs were systematically captured, the severity of the AEs were graded using Common Toxicity Criteria (CTC) of the National Cancer Institute in 34 (22.8%), WHO toxicity scale in 5 (3.4%), Division of AIDS Table for grading severity in 2 (1.3%), and the grading standards adopted was not described or unclear in the remaining 108 (72.5%) studies (See S1 Data).

### Dose testing in amphotericin B formulation arms

History of hypersensitivity to the study drug was an exclusion criteria in 98 (28.2%) treatment arms of which 57 arms (58.2%, 57/98) tested amphotericin B (any formulation or in combination with other drugs). Out of 165 treatment arms (n = 14,195 patients) with amphotericin B (any formulation or combination), dose testing prior to initiation of treatment regimen was reported in 38 (23.0%) arms (n = 4,594 patients). Two patients developed allergic reactions during a test infusion with 1 mg of L-AmB and were discontinued from the study. Using a random effects meta-analysis of single proportion using logistic transformation, the rate of allergic reactions leading to treatment discontinuation after dose testing was estimated at 0.4 cases [95% CI: 0.1–1.7] per 1,000 tested participants (I^2^ = 0.0%; 38 arms, 4,594 patients).

### Serious adverse events (SAEs)

A total of 804 SAEs could be extracted from the studies included in the analysis. In two studies, 11 SAEs were reported at the study level and could not be assigned to specific treatment arms leaving 793 SAEs, which could be identified at the treatment arm level. Of the latter, a total of 370 (46.7%) SAEs were clearly reported to occur during the first 30 days since the initiation of treatment, 41 (5.2%) occurred between the study follow-up days 31 through 180, 33 (4.2%) occurred after the completion of the treatment (after 30 days without a clear precision of the time range), 3 (0.4%) occurred between 181 through 360 days, and the time of occurrence was not clear in the remaining 346 (43.6%) (S1 Data). Of the 793 SAEs, there were 311 (39.2%) among patients treated with PA, 131 (16.5%) among those treated with miltefosine, 80 (10.1%) among those treated with amphotericin b deoxycholate; a further breakdown is presented in supplemental file (S1 Data). In total, 288 (36.3%) SAEs lacked any specific description, 105 (13.2%) were cardiac disorders, 83 (10.5%) were infections and infestations, 71 (8.9%) were blood and lymphatic disorders, and 70 (8.8%) were gastrointestinal disorders. The most commonly reported SAEs were cardiac disorders following PA (25.4%, 79/311), gastrointestinal disorders following miltefosine (27.5%, 36/131), and anaemia and blood disorders following multiple dose L-AmB (27.3%, 12/44). Further description on the SAEs is presented in supplemental file (S1 Data). Of the 793 SAEs identified at study arm levels, 428 (54.0%) led to death.

### Death reporting

There was a total of 439 deaths of which 428 could be identified to the specific treatment arm under study. Of the latter, 285 (66.6%) deaths were clearly described as occurring during the first 30 days of enrolment, 70 (16.4%) deaths occurred after completion of the treatment, i.e. between day 31 and the end of the study follow-up. In the remaining 73 (17.1%), the time of the occurrence of deaths could not be clearly ascertained. There were 60 (14.0%) deaths which were described as related to or possibly/probably/might be related to the drug administered (Table 2). Further description of time and causes of deaths are reported in supplemental file (S1 Data).

**Table 2:** Description of deaths adjudicated as related to or probably/possibly related to study drug (n = 60).

Count ^a^	Study	Country	Drug	Dosage	Time of death (day)	Description of SAE
1	Sundar-2011b	India	AMBd	1 mg/kg AMBd by IV infusion over 6h in 5% dextrose on alternate days for 30 days (total dose of 15 mg/kg)	0 to 30	Cardiac infarction
2	Thakur-2004a	India	PA	20 mg/kg/day for 28 days	21 to 28	Serious ventricular arrhythmia
3	Thakur-2004a	India	PA	20 mg/kg/day for 28 days	21 to 28	Cardiac arrest
4	Jha-2005	India	Sitamaquine	2.5 mg/kg/day for 28 days	11	Elevated hepatic enzymes
5	Thakur-1998b	India	PA	20 mg/kg for 30 days (to a maximum daily dose of 850 mg)	10	Cardiotoxicity
6	Thakur-1998b	India	PA	20 mg/kg for 30 days (to a maximum daily dose of 850 mg)	14	Cardiotoxicity
7	Thakur-1998b	India	PA	20 mg/kg for 30 days (to a maximum daily dose of 850 mg)	15	Cardiotoxicity
8	Thakur-1998b	India	PA	20 mg/kg for 30 days (to a maximum daily dose of 850 mg)	18	Cardiotoxicity
9	Sundar-2012	India	Miltefosine	2.5 mg/kg for children under 12, 100 mg if >25 kg, and 50 mg if <25 kg per day for 28 days	3rd week of treatment	Repeated vomiting and severe pancytopenia
10	Sundar-2000b	India	PA	20 mg/kg/day for 30 days (without any upper dose limit)	Not clear	Cardiotoxicity
11	Sundar-2000b	India	PA	20 mg/kg/day for 30 days (without any upper dose limit)	Not clear	Cardiotoxicity
12	Sundar-2000b	India	PA	20 mg/kg/day for 30 days (without any upper dose limit)	Not clear	Cardiotoxicity
13	Sundar-2000b	India	PA	20 mg/kg/day for 30 days (without any upper dose limit)	Not clear	Cardiotoxicity
14	Sundar-2000b	India	PA	20 mg/kg/day for 30 days (without any upper dose limit)	Not clear	Cardiotoxicity
15	Sundar-2000b	India	PA	20 mg/kg/day for 30 days (without any upper dose limit)	Not clear	Cardiotoxicity
16	Sundar-2000b	India	PA	20 mg/kg/day for 30 days (without any upper dose limit)	Not clear	Cardiotoxicity
17	Sundar-2000b	India	PA	20 mg/kg/day for 30 days (without any upper dose limit)	Not clear	Cardiotoxicity
18	Sundar-2000b	India	PA	20 mg/kg/day for 30 days (without any upper dose limit)	Not clear	Cardiotoxicity
19	Sundar-2000b	India	PA	20 mg/kg/day for 30 days (without any upper dose limit)	Not clear	Cardiotoxicity
20	Sundar-2000b	India	PA	20 mg/kg/day for 30 days (without any upper dose limit)	Not clear	Cardiotoxicity
21	Sundar-2000b	India	PA	20 mg/kg/day for 30 days (without any upper dose limit)	Not clear	Cardiotoxicity
22	Sundar-2000b	India	PA	20 mg/kg/day for 30 days (without any upper dose limit)	Not clear	Cardiotoxicity
23	Sundar-2000b	India	PA	20 mg/kg/day for 30 days (without any upper dose limit)	Not clear	Cardiotoxicity
24	Sundar-2000b	India	PA	20 mg/kg/day for 30 days (without any upper dose limit)	Not clear	Cardiotoxicity
25	Sundar-2000b	India	PA	20 mg/kg/day for 30 days (without any upper dose limit)	Not clear	Cardiotoxicity
26	Sundar-2000b	India	PA	20 mg/kg/day for 30 days (without any upper dose limit)	Not clear	Cardiotoxicity
27	Sundar-2000b	India	PA	20 mg/kg/day for 30 days (without any upper dose limit)	Not clear	Cardiotoxicity
28	Sundar-2000b	India	PA	20 mg/kg/day for 30 days (without any upper dose limit)	Not clear	Cardiotoxicity
29	Sundar-2007a	India	AMBd	1 mg/kg IV every other day for 30 days	Not clear	Gastroenteritis/diarrhoea
30	Sundar-2007a	India	Paromomycin	11 mg/kg/day for 21 days	After 2 doses	Increased aspartate aminotransferase levels
31	Rahman-2011	Bangladesh	Miltefosine	2.5mg/kg for children under 12, 100mg if >25kg, and 50 mg if <25kg per day for 28 days	21	Diarrhoea
32	Musa-2012	Sudan, Ethiopia, Kenya, Uganda	PA	SSG 20 mg/kg/day for 30 days either IM or IV	11	Cardiotoxicity
33	Musa-2012	Sudan, Ethiopia, Kenya, Uganda	PA	SSG 20 mg/kg/day for 30 days either IM or IV	During initial hospitalisation	Renal impairment
34	Musa-2012	Sudan, Ethiopia, Kenya, Uganda	PA	SSG 20 mg/kg/day for 30 days either IM or IV	During initial hospitalisation	Renal impairment
35	Thakur-1984a	India	Pentamidine	4 mg/kg every second or third day to a total of 15 injections	14	Severe anorexia
36	Thakur-1984a	India	Pentamidine	4 mg/kg every second or third day to a total of 15 injections	3	Hepatic failure
37	Thakur-1984a	India	Pentamidine	4 mg/kg every second or third day to a total of 15 injections	During therapy	Hypersensitivity to drug
38	Thakur-1984a	India	Pentamidine	4 mg/kg every second or third day to a total of 15 injections	During therapy	Hypersensitivity to drug
39	Thakur-1984a	India	Pentamidine	4 mg/kg every second or third day to a total of 15 injections	During therapy	Hypersensitivity to drug
40	Sundar-2014	India	Amphotericin b fat/lipid/colloid/cholesterol	15 mg/kg single infusion	2	Severe diarrhoea
41	Wasunna-2016	Kenya, Sudan	L-AmB + PA	L-AmB 10 mg/kg on day 1 infused in 5% dextrose over 1–2 hours + 10 days of SSG at 20 mg/kg on days 2–11	20	Severe anaemia leading to death
42	Romero-2017	Brazil	PA	20 mg Sb+ 5/kg/day intravenous (IV) for 20 days, with a maximum of 1,215 mg pentavalent antimony (Sb+5; three 5 mL vials) per day	11	Respiratory and hemodynamic worsening and death due to presumed sepsis
43	Pandey-2016	India	Miltefosine	100 mg in two divided doses for patients > 12 years of age or body weight ≥ 25 kg OR 50 mg or 2.5 mg per kg body weight for patients ≤ 12 years or body weight < 25 kg	Not clear	Extreme diarrhoea
44	Pandey-2016	India	Miltefosine	100 mg in two divided doses for patients > 12 years of age or body weight ≥ 25 kg OR 50 mg or 2.5 mg per kg body weight for patients ≤ 12 years or body weight < 25 kg	Not clear	Acute pancreatitis
45 to 60	Kimutai-2017	Sudan, Ethiopia, Kenya, Uganda	PA + Paromomycin	IV or IM PA at 20 mg/kg/day and IM paromomycin at 15 mg/ kg/day for 17 days	Not clear	“Of the 16 deaths considered related to SSG-PM treatment, anaemia (n = 4), sudden death (n = 2) and renal-related AEs were the leading causes”

SSG = sodium stibogluconate; L-AmB = Liposomal amphotericin B; PA = pentavalent antimony; AMBd = amphotericin B deoxycholate; IV = intravenous; IM = intramuscular; PM = Paromomycin; AEs = Adverse events

^a^ A case of serious adverse event described in Wassuna-2005 [10] that was considered as suspected or probably related to study drug (sitamaquine) is not included in this table as the primary cause of death was due to pulmonary arteriole thromboembolism (although the patient experienced SAEs of glomerulonephritis and chronic renal failure). Further details on the studies described are presented in S1 Data.

### Estimate of incidence rate of death (IRD) within 30 days of treatment initiation

The overall IRD within the first 30 days of treatment initiation was 0.068 [95% CI: 0.041–0.114; I^2^ = 81.4%; 325 arms, 31,706 patients] per 1,000 person-days. The 95% prediction interval (PI) for a future study ranged from 0.001 to 2.779 per 1,000 person-days. After stratifying by the region, the rates were 0.628 [95% CI: 0.386–1.021; I^2^ = 82.5%; 56 arms, 7,291 patients] in Eastern Africa, 0.041 [95% CI: 0.021–0.081; I^2^ = 68.1%; 215 arms; 23,052 patients] in the Indian Subcontinent, 0.018 [95% CI: 0.000–6.514; I^2^ = 85.0%; 18 arms; 397 patients] in the Mediterranean region, and 0.118 [95% CI: 0.029–0.473; I^2^ = 0.0%; 17 arms; 563 patients] in Southern America (Table 3).

**Table 3:** Incidence rate of death per 1,000 person-days within the first 30 days of treatment initiation.

	n/P/d	Random effects analysis [95% confidence interval]	I^2^	95% prediction interval	Bias adjusted estimates ^a^ [95% confidence interval]
**Amphotericin B deoxycholate**					
India Subcontinent	44/4945/22	0.079 [0.028–0.222]	49.3%	0.007–0.804	0.365 [0.267–0.499]
Southern America	2/95/0	-	-	-	-
Eastern Africa	1/210/10	-	-	-	-
Overall	47/5250/32	0.069 [0.023–0.207]	66.5%	0.003– .318	1.114 [0.752–1.649]
**Amphotericin b (fat/lipid/colloid/cholesterol)**					
India Subcontinent	23/1187/2	0.056 [0.014–0.224]	0.0%	0.014–0.224	0.597 [0.343–1.039]
Mediterranean	3/50/0	-	-	-	-
South America	3/30/0	-	-	-	-
Overall	29/1267/2	0.052 [0.013–0.210]	0.0%	0.013–0.210	0.694 [0.422–1.143]
**L-AmB (Single dose)**					
India Subcontinent	15/3231/2	0.017 [0.002–0.129]	27.1%	0.001–0.225	0.268 [0.125–0.572]
Eastern Africa	1/40/0	-	-	-	-
Overall	16/3271/2	0.017 [0.002–0.128]	26.1%	0.001–0.219	0.273 [0.134–0.558]
**L-AmB (Single dose) combination regimen**					
India Subcontinent	7/833/1	0.040 [0.005–0.284]	0.0%	0.005–0.284	0.219 [0.082–0.586]
South America	1/112/1	-	-	-	-
Overall	8/945/2	0.070 [0.017–0.282]	0.0%	0.017–0.282	0.233 [0.097–0.561]
**L-AmB (Multiple doses)**					
India Subcontinent	17/654/2	0.102 [0.025–0.407]	0.0%	0.025–0.407	0.752 [0.398–1.421]
Eastern Africa	6/195/7	1.040 [0.267–4.050]	63.0%	0.070–15.346	3.315 [1.359–8.087]
Mediterranean	13/276/1	0.063 [0.000–19.661]	47.1%	0.000–33.473	1.112 [0.530–2.333]
Multi-Regional	9/185/0	-	-	-	-
South America	4/141/0	-	-	-	-
Not stated	1/10/0	-	-	-	-
Overall	49/1451/10	0.068 [0.010–0.435]	68.9%	0.001–4.221	2.470 [1.791–3.406]
**L-AmB (multiple) combination regimen**					
Eastern Africa	2/90/2	-	-	-	-
India Subcontinent	2/318/0	-	-	-	-
Overall	4/408/2	0.147 [0.023–0.947]	36.4%	0.011–1.888	0.388 [0.125–1.204]
**Miltefosine**					
Eastern Africa	3/371/6	0.539 [0.242–1.199]	0.0%	0.242–1.199	0.643 [0.306–1.350]
India Subcontinent	28/4379/10	0.076 [0.041–0.141]	0.0%	0.041–0.141	0.070 [0.040–0.123]
Overall	31/4750/16	0.090 [0.036–0.225]	44.1%	0.011–0.732	0.336 [0.184–0.611]
**Miltefosine + Paromomycin**					
India Subcontinent	3/813/0	-	-	-	-
Overall	3/813/0	-	-	-	-
**Pentavalent Antimony**					
Eastern Africa	24/2187/132	1.311 [0.784–2.192]	81.0%	0.204–8.422	2.741 [1.880–3.996]
India Subcontinent	31/3941/21	0.025 [0.001–0.334]	84.4%	0.000–4.482	0.500 [0.321–0.777]
Mediterranean	2/71/3	-	-	-	-
Central Asia	9/211/1	0.166 [0.023–1.181]	0.0%	0.023–1.181	0.895 [0.372–2.150]
South America	2/163/1	-	-	-	-
Not stated	1/23/0	-	-	-	-
Overall	69/6596/158	0.215 [0.099–0.466]	88.7%	0.006–7.820	2.478 [1.977–3.105]
**PA combination regimen**					
India Subcontinent	15/574/6	0.038 [0.000–4.468]	80.7%	0.000–28.473	1.021 [0.584–1.786]
Eastern Africa	5/3654/37	0.323 [0.126–0.826]	52.9%	0.066–1.575	0.468 [0.221–0.989]
Overall	20/4228/43	0.203 [0.054–0.762]	65.2%	0.016–2.554	0.666 [0.408–1.087]
**Paromomycin**					
India Subcontinent	10/1131/3	0.077 [0.007–0.799]	8.1%	0.006–0.888	0.269 [0.131–0.551]
Eastern Africa	4/382/1	0.087 [0.012–0.619]	0.0%	0.012–0.619	0.290 [0.084–1.002]
Overall	14/1513/4	0.088 [0.033–0.234]	0.0%	0.033–0.234	0.274 [0.147–0.510]
**Pentamidine**					
India Subcontinent	8/603/13	0.341 [0.072–1.605]	74.0%	0.015–7.482	1.911 [1.062–3.438]
Overall	8/603/13	0.341 [0.072–1.605]	74.0%	0.015–7.482	1.911 [1.062–3.438]
**Sitamaquine**					
India Subcontinent	5/161/1	0.207 [0.029–1.469]	0.0%	0.029–1.469	0.673 [0.217–2.087]
Eastern Africa	7/113/0	-	-	-	-
South America	5/22/0	-	-	-	-
Overall	17/296/1	0.112 [0.015–0.799]	0.0%	0.015–0.799	1.593 [0.829–3.063]
**All data**	325/31706/285	0.068 [0.040–0.114]	81.4%	0.001–2.779	1.358 [1.170–1.577]

* n = number of study arms combined; d = total number of deaths within first 30 days of treatment initiation; P = Total number of treated patients from all the arms which contributed to the meta-analysis; rates are expressed per 1,000 person-days; L- AmB = Liposomal amphotericin B; PA = pentavalent antimony; CI = Confidence Interval; I^2^ = measure of heterogeneity in the results; the incidence rate of death (IRD) is estimated using a random effects Poisson regression

^a^ The estimates of incidence rates adjusted for potential publication biases derived using Copas selection model

### Estimate of the IRD within 30-day of treatment initiation stratified by enrolment of patients of HIV co-infections

There was a significant sub-group difference in the IRD when accounting for studies allowed inclusion of patients with HIV co-infection in a meta-regression (I^2^ = 75.4%; *P*-value<0.001). In 21 study arms that also enrolled patients with HIV co-infections; the corresponding IRD was 0.575 [95% CI: 0.244–1.352; I^2^ = 91.9%; 21 arms; 5,250 patients]. In 160 study arms, where HIV co-infection was clearly an exclusion criteria, the overall IRD was 0.043 [95% CI: 0.020–0.090; I^2^ = 62.5%; 16,772 patients]; mean difference in IRD between the two groups was 0.532 [95% CI: 0.038–1.026] per 1,000 person-days. In study arms which clearly excluded HIV co-infections, the IRD was 0.042 [95% CI: 0.019–0.094; 117 arms; I^2^ = 62.4%] in the ISC and 0.061 [95% CI: 0.002–2.236; 15 arms; I^2^ = 73.5%] in EA. See supplemental files (S1 Table and S1 Data) for further details.

### Estimate of the IRD within 30-day of treatment initiation stratified by drug regimens

The IRD was 0.069 [95% CI: 0.023–0.207;I^2^ = 66.5%; 47 arms; 5,250 patients] per 1,000 person-days for amphotericin b deoxycholate; 0.052 [95% CI: 0.013–0.210; I^2^ = 0.0%; 29 arms; 1,267 patients] for amphotericin b with fat/lipid/colloid/cholesterol; 0.017 [95% CI: 0.002–0.128; I^2^ = 26.1; arms; 3,271 patients] for single-dose L-AmB, and 0.068 [95% CI: 0.010–0.435; I^2^ = 68.9%; 49 arms; 1,451 patients] for multiple-dose L-AmB. In studies which tested L-AmB in a combination regimen with another drug, the corresponding rates were 0.070 [95% CI: 0.017–0.282; 8 arms; 945 patients] and 0.147 [95% CI: 0.023–0.947; 4 arms; 408 patients] respectively for the single and multiple dose L-AmB regimens. The estimated rates were 0.215 [95% CI: 0.099–0.466; I^2^ = 88.7%; 69 arms; 6,596 patients] for pentavalent antimony, and 0.203 [95% CI: 0.054–0.762; I2 = 65.2%; 20 arms; 4,228 patients] when PA was administered in 44.1%; 31 arms; 4,750 patients]. After stratifying by region, the IRDs for multiple doses of L-AmB, pentavalent antimony, and miltefosine were higher in Eastern Africa than in the Indian Subcontinent (Table 3).

### Adjustment for potential publication biases

**Fig 2:**
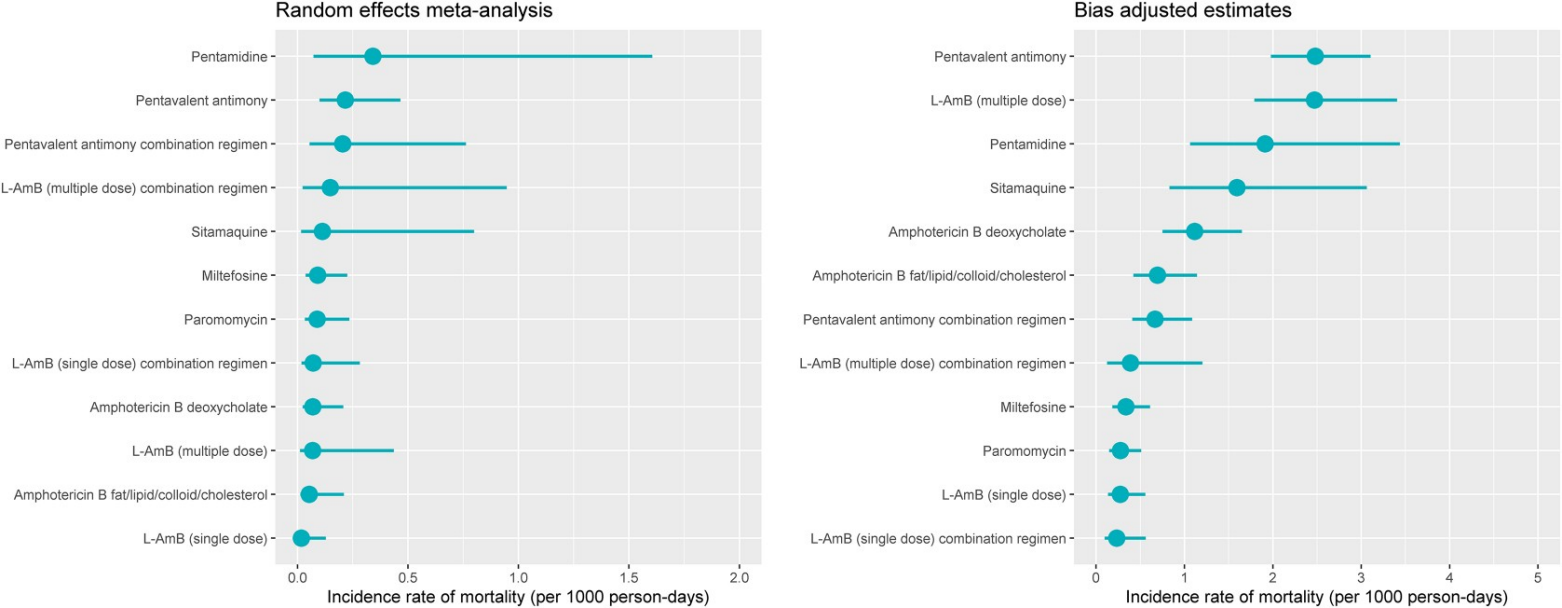
Incidence rate of death within 30 days of treatment initiation. The left-hand panel presents the pooled estimate of incidence rate of death from random effects meta-analysis with 95% confidence interval. The right-hand panel presents the estimate and 95% confidence interval from analysis which adjusted for potential publication bias; L-AmB = Liposomal amphotericin B; PA = pentavalent antimony; AMBd = amphotericin B deoxycholate.

The incidence rates of deaths were re-estimated to account for potential publication biases; the adjusted rates were 2.6 to 36-fold larger compared to the unadjusted rates (Table 3 and Fig 2).

### Sensitivity analyses and risk of bias assessment

In a worst-case scenario analysis, that considered all the deaths with unclear time as occurring within the first 30 days of treatment initiation, the overall estimated incidence rate of death was 0.069 [95% CI: 0.041–0.116; I^2^ = 82.1%] per 1,000 person-days (S2 Table). The rates were estimated separately for randomised and non-randomised studies; the estimated rate was 0.046 [95% CI: 0.020–0.103] for the randomised studies and 0.129 [95% CI: 0.067–0.246] for non-randomised studies (*P*-value: 0.0011 for test of sub-group differences) (S3 Table). The IRD estimates were higher in studies considered to be at high risk of bias due to incomplete outcome reporting for both randomised and non-randomised studies (S4–S7 Tables). Similarly, risk of bias was also assessed in an eligible study that was initially excluded due to unavailability of full text [38]. The study tested miltefosine regimen in 160 children and reported 2 deaths within a week of treatment initiation. The IRD within 30 days was estimated at 0.416 [95% CI: 0.056–1.505]; the point estimate for IRD was larger but the 95% CI overlapped with the estimates for miltefosine for the Indian Subcontinent (Table 3).

## Discussion

From this systematic review of all published antileishmanial efficacy clinical trials conducted in the last 4 decades, we report several findings directly relevant to researchers, field workers and policymakers regarding different aspects of drug safety.

Hypersensitivity to the study drug is one of the major safety concerns in antileishmanial chemotherapies. Case reports of anaphylactic shock following amphotericin B has been well-documented [39]. To obviate this, manufacturers recommend that patients are given a small test dose of L-AmB before the commencement of the full treatment course and hypersensitivity to L-AmB is often an exclusion criterion in clinical trials [40]. From 165 treatment arms with amphotericin B formulations included in this review, the estimated rate of discontinuations from the study after administration of a test dose was 0.4 [95% CI: 0.1–1.7] per 1,000 tested participants. This might, however, underestimate the true rate, since a history of drug hypersensitivity was an exclusion criteria in 57 arms (34.5%) that tested amphotericin B formulations, and reporting of dose testing information was available only in 38 (23%) study arms with this regimen.

There was a total of 793 SAEs that were reported at the study arm level; the majority of the descriptions regarding the cause were non-specific and the time of occurrence was missing in just over half, which precluded meta-analysis. As with the SAEs, the reporting of fatal outcomes was also inconsistent with the time of occurrence of death not documented in nearly a fifth of all reported deaths. Overall, mortality was an extremely rare event with the rate estimated at 0.068 [95% CI: 0.041–0.114; 95% PI: 0.001–2.779] per 1,000 person-days at risk with substantial statistical heterogeneity in results (I^2^ = 81.4%).

Some of the heterogeneity was explained by geographical region as the overall estimated rate was much higher in Eastern Africa (0.628 [95% CI: 0.368–1.021]) than in the Indian Subcontinent (0.041 [95% CI: 0.021–0.081]) (Table 3). For a given drug regimen, the estimates of the mortality rate across the regions were comparable for all the drug regimens with the exception of pentavalent antimony, multiple dose L-AmB regimen, and miltefosine, for which the estimated incidence rates were much higher in Eastern Africa (EA) than in the Indian Subcontinent (ISC). The larger rates observed in EA was partly explained by the clinical differences in patients enrolled as the studies from EA were more likely to enrol patients with HIV co-infectionns compared to studies from the ISC (16% in EA compared to 0.5% in the ISC); and the estimated mortality rate was greater than 10-fold higher in study arms which indicated patients with HIV co-infections were eligible for enrolment. This is consistent with published literature that the case fatality rate is much higher in HIV co-infected individuals compared to VL infections alone [41]. In patients with HIV infections, VL manifests as an opportunistic infection with disease severity exacerbated due to the depleted immune response, therefore with higher risk of poor treatment outcome and death. There is also an increased complexity in terms of case management as these patients are at an increased risk of drug toxicity due to interactions between antiretroviral and antileishmanial drugs, though these factors have not been extensively studied [42].

This review has several limitations. First, information regarding the time and causes of death and the relationship to the study drugs were found to be poorly described in the existing literature. This inconsistency in reporting made it difficult to interpret the data and prevented a direct comparison of the incidence rate of deaths between different regimens. We therefore carried out sensitivity analyses to explore the worst-case scenario where all the deaths with an unclear time of occurrence were considered as occurring within the treatment period; the estimates of IRD obtained from the sensitivity analyses were similar to the results reported in Table 3 (See S5 and S7 Tables). Like with mortality data, extraction of data on SAEs was difficult because most of the description were either not specific or the time when SAEs occurred was not available in just over a third of them. In particular, the underlying denominators were not clear as some patients could have experienced multiple SAEs. Second, despite there being only a few classes of antileishmanial drugs, there were many different formulations and dosages of amphotericin B or antimonies administered across the included studies. The multiplicity of drug regimens posed analytical challenges for evidence synthesis when using aggregated information from publications. Third, mortality is a rare event and several trials had no reported deaths, leading to a statistical problem of structural zeros. How best to handle such structural zeros in meta-analysis has remained a contentious issue in the statistical literature [30,43–46]. The results presented in this review are derived from a random effects meta-analysis using Poisson regression model, following the recommendations of Spittal et al. [30]. Fourth, clinical aspects of the disease such as severity of illness and unresponsiveness of the parasites were not considered as data on these parameters could not be reliably extracted. Similarly, clinical trials by nature cannot detectsome SAEs like birth/congenital defects which can only be captured through prospective cohort studies/pregnancy registries. Finally, the Copas selection model indicated existence of publication bias as the adjusted estimates were larger than the unadjusted rates (Table 3 and Fig 2). The estimated rates were also affected by the heterogeneity in study design and by the risk of bias status in different domains (S5 and S7 Tables). The estimated rates should therefore need a cautious interpretation with these limitations taken into considerations.

Despite these limitations, this review reports several important findings which can help field workers in the assessment of the safety of antileishmanial therapies and guide further research with the data presented in this review serving as a baseline rate. This review has also found that reporting of safety outcomes in VL trials remains inconsistent, a finding in agreement to a previously published research [47]. In just over two-third of the studies included, the standards adopted for the gradation of the adverse events and serious adverse events were not mentioned. This suggests a need for clear and transparent reporting of the methodology used in safety assessment and warrants an adoption of recognised standards for reporting of safety outcomes such as Medical Dictionary for Regulatory Activities (MedDRA) or the Common Terminology Criteria for Adverse Events [48,49].

A carefully planned individual participant data (IPD) meta-analysis will ameliorate some of the limitations identified in this aggregated data meta-analysis and will allow the generation of a more robust estimate by amalgamating the current evidence base to the highest standard. The efforts of assembling and standardising IPD are ongoing under a data platform championed by the Infectious Diseases Data Observatory (see https://www.iddo.org/research-themes/visceral-leishmaniasis). This is of utmost importance as the current research and development drug pipeline is limited, and improvement of safety reporting will also benefit future surveillance for new drugs that are under development [18]. Another factor to consider is the delivery of treatment at the “point of care” in national programmes which are often ill equipped in rural areas and lack trained staff to administer antileishmanial drugs. This real-life situation is much different than carefully planned clinical trials with mechanisms to monitor and address the incidence of adverse events. A better understanding of the safety and tolerability profile of existing treatment regimens is therefore critical for the assessment of therapeutic risk-benefit balance to facilitate judicious use of treatment. This can, in turn, prolong the shelf-life of the current therapeutic arsenal at the frontline of elimination programmes and aid in combating drug resistance.

In conclusion, the overall expected incidence rate of death in the first 30-days after treatment initiation was a rarely reported event in clinical trials with the rate estimated at 0.068 per 1,000 person-days at risk [95% prediction interval: 0.001–2.779]. The estimated rates were higher in studies that allowed enrolment of patients with HIV co-infections and in studies from Eastern Africa compared to the Indian Subcontinent. The analysis of safety data was significantly inhibited by poor reporting and warrants a better and standardised reporting.

## Supporting information

S1 Text PRISMA checklist. (DOC)

S2 Text Literature search strategy. (DOCX)

S3 Text Comparison of overall incidence rate of death between two groups. (DOCX)

S1 Table Incidence rate of death in studies that allowed inclusion of patients with HIV co-infections. (DOCX)

S2 Table Sensitivity analysis assuming worst case scenario. (DOCX)

S3 Table Incidence rate of death stratified by randomisation status. (DOCX)

S4 Table Assessment of risk of bias in randomised studies included in the review. (DOCX)

S5 Table Incidence rate of death stratified by risk of bias status in randomised studies. (DOCX)

S6 Table Assessment of risk of bias in studies other than randomised allocation of patients. (DOCX)

S7 Table Incidence rate of death stratified by risk of bias status in studies other than randomised allocation of patients. (DOCX)

S1 Data Dataset used for analysis. (XLSX)
