# Supplementary material for: Serious adverse events following treatment of visceral leishmaniasis: A systematic review and meta-analysis
Source: PLoS Negl Trop Dis. 2021 Mar 29;15(3):e0009302. doi: 10.1371/journal.pntd.0009302 (PMC8031744; doi:10.1371/journal.pntd.0009302)
Supplement: S2 Text — (DOCX) [file pntd.0009302.s002.docx]

**S2 Text:** **Literature search strategy**

We updated the Bush et al. (2017) systematic review, which had reported 162 studies (145 published) published between 1980 and 2015 by screening articles published between 1-Jan-2016 to 2-May-20192 (inclusive)[1]. Search strategies run on 02/05/19. Details on each of the databases searched are presented below:

### **Pubmed**

((((((((randomized controlled trial[pt] OR controlled clinical trial[pt] OR randomized[tiab] OR placebo[tiab] OR clinical trials as topic[mesh:noexp] OR randomly[tiab] OR trial[ti] NOT (animals[mh] NOT humans [mh]))) AND Clinical Trial[ptyp])) AND (((("Leishmaniasis, Visceral"[Mesh]) OR visceral leishmaniasis[Title/Abstract]) OR kala azar[Title/Abstract]) OR black fever[Title/Abstract]))) OR ((((((((((((((("Pentamidine"[Mesh]) OR "liposomal amphotericin B" [Supplementary Concept]) OR "Amphotericin B"[Mesh]) OR "Paromomycin"[Mesh]) OR "miltefosine" [Supplementary Concept]) OR "Sodium"[Mesh]) OR "8-aminoquinoline" [Supplementary Concept]) OR "Ketoconazole"[Mesh]) OR "Azoles"[Mesh]) OR "Allopurinol"[Mesh]) OR ( "Atovaquone"[Mesh] OR "atovaquone, proguanil drug combination" [Supplementary Concept] ))) OR ((pentamidine[Title/Abstract] OR ambisome[Title/Abstract] OR amphotericin[Title/Abstract] OR paromomycin[Title/Abstract] OR miltefosine[Title/Abstract] OR pentavalent[Title/Abstract] OR sodium[Title/Abstract] OR sitamaquine[Title/Abstract] OR azole*[Title/Abstract] OR allopurinol[Title/Abstract] OR atovaquone[Title/Abstract] OR ketoconazole[Title/Abstract] OR fluconazole[Title/Abstract] OR metronidazole[Title/Abstract])))) AND (((("Leishmaniasis, Visceral"[Mesh]) OR visceral leishmaniasis[Title/Abstract]) OR kala azar[Title/Abstract]) OR black fever[Title/Abstract])))) NOT (((animals not humans)))

Filters activated: Publication date from 2016/01/01 to 2019/12/31

This search strategy uses the PubMed RCT filter for sensitivity- and precision-maximising version (2008). <https://work.cochrane.org/pubmed>

### **Embase**

Date: 1974 to present

--------------------------------------------------------------------------------

1 exp visceral leishmaniasis/ (9032)

2 "black fever".ti,ab. (16)

3 "kala azar".ti,ab. (2138)

4 "visceral leishmaniasis".ti,ab. (8713)

5 1 or 2 or 3 or 4 (11608)

6 exp randomized controlled trial/ (546363)

7 Controlled clinical study/ (461844)

8 Random$.ti,ab. (1401563)

9 randomization/ (82089)

10 intermethod comparison/ (246778)

11 placebo.ti,ab. (286120)

12 (compare or compared or comparison).ti. (477335)

13 ((evaluated or evaluate or evaluating or assessed or assess) and (compare or compared or comparing or comparison)).ab. (1903401)

14 (open adj label).ti,ab. (70475)

15 ((double or single or doubly or singly) adj (blind or blinded or blindly)).ti,ab. (217393)

16 double blind procedure/ (159727)

17 parallel group$1.ti,ab. (23261)

18 (crossover or cross over).ti,ab. (97631)

19 ((assign$ or match or matched or allocation) adj5 (alternate or group$1 or intervention$1 or patient$1 or subject$1 or participant$1)).ti,ab. (302405)

20 (assigned or allocated).ti,ab. (355650)

21 (controlled adj7 (study or design or trial)).ti,ab. (316500)

22 (volunteer or volunteers).ti,ab. (233422)

23 trial.ti. (267888)

24 6 or 7 or 8 or 9 or 10 or 11 or 12 or 13 or 14 or 15 or 16 or 17 or 18 or 19 or 20 or 21 or 22 or 23 (4285150)

25 5 and 24 (1387)

26 (pentamidine or ambisome or amphotericin or paromomycin or miltefosine or pentavalent or sodium or sitamaquine or azole* or allopurinol or atovaquone or ketoconazole or fluconazole or metronidazole).mp. (957377)

27 5 and 26 (3490)

28 25 or 27 (4481)

29 (exp animal/ or animal.hw. or nonhuman/) not (exp human/ or human cell/ or (human or humans).ti.) (6108726)

30 28 not 29 (3496)

31 30 (3496)

32 limit 31 to yr="2016 -Current" (549)

This search strategy uses the Cochrane RCT filter for Embase

[https://www.cochranelibrary.com/central/central‐creation](https://www.cochranelibrary.com/central/central-creation)

### **Scopus**

( ( TITLE-ABS-KEY ( "visceral leishmaniasis" OR "kala azar" OR "black fever" ) ) AND ( ( TITLE-ABS-KEY ( random* OR rct OR placebo OR allocat* OR crossover* OR "cross over" OR trial OR ( doubl* W/1 blind* ) OR ( singl* W/1 blind* ) ) ) OR ( TITLE-ABS-KEY ( control* W/1 trial* ) ) OR ( TITLE-ABS-KEY ( cohort* ) ) ) ) OR ( ( TITLE-ABS-KEY ( "visceral leishmaniasis" OR "kala azar" OR "black fever" ) ) AND ( TITLE-ABS-KEY ( pentamidine OR ambisome OR amphotericin OR paromomycin OR miltefosine OR pentavalent OR sodium OR sitamaquine OR azole* OR allopurinol OR atovaquone OR ketoconazole OR fluconazole OR metronidazole ) ) ) AND ( EXCLUDE ( EXACTKEYWORD , "Animals" ) OR EXCLUDE ( EXACTKEYWORD , "Animal" ) OR EXCLUDE ( EXACTKEYWORD , "Animal Experiment" ) OR EXCLUDE ( EXACTKEYWORD , "Mouse" ) OR EXCLUDE ( EXACTKEYWORD , "Mice" ) OR EXCLUDE ( EXACTKEYWORD , "Dogs" ) ) AND ( LIMIT-TO ( PUBYEAR , 2019 ) OR LIMIT-TO ( PUBYEAR , 2018 ) OR LIMIT-TO ( PUBYEAR , 2017 ) OR LIMIT-TO ( PUBYEAR , 2016 ) )

### **Web of Science Core Collection**

1. TOPIC: ("visceral leishmaniasis" OR "kala azar" OR "black fever")
2. TOPIC: (random* OR rct OR placebo OR allocat* OR crossover* OR "cross over" OR trial OR (doubl* near/1 blind*) OR (singl* near/1 blind*))
3. TOPIC: (control* near/1 trial*)
4. TOPIC: (cohort*)
5. #4 OR #3 OR #2
6. #5 AND #1
7. TOPIC: (pentamidine OR ambisome OR amphotericin OR paromomycin OR miltefosine OR pentavalent OR sodium OR sitamaquine OR azole* OR allopurinol OR atovaquone OR ketoconazole OR fluconazole OR metronidazole)
8. #7 AND #1
9. #8 OR #6
10. #8 OR #6
11. Refined by: PUBLICATION YEARS: ( 2019 OR 2018 OR 2017 OR 2016 )

### **Cochrane Central Register of Controlled Trials**

Issue 5 of 12, May 2019

#1 MeSH descriptor: [Leishmaniasis, Visceral] explode all trees 144

#2 "visceral leishmaniasis" 230

#3 "kala azar" 113

#4 "black fever" 1

#5 #1 or #2 or #3 or #4 276

Custom Range: 2016 to 2019

### **World Health Organization Global Index Medicus**

<http://www.globalhealthlibrary.net/php/index.php?lang=en>

tw:(tw:(("visceral leishmaniasis" OR "kala azar" OR "black fever") ) AND (instance:"ghl") AND ( year_cluster:("2016" OR "2017" OR "2018" OR "2019"))) AND (instance:"ghl")

### **ClinicalTrials.gov**

Advanced Search <https://clinicaltrials.gov/ct2/search/advanced>?

Condition or disease: visceral leishmaniasis OR kala azar OR black fever

2016 – date

### **WHO International Clinical Trials Registry Platform**

<http://apps.who.int/trialsearch/>

visceral leishmaniasis OR kala azar OR black fever – Trials at ALL stages

2016 – date

**Summary of Search Results**

The updated screening identified an additional 19 studies which were merged with the previously identified 162 studies. After removing the duplicates, we finally had 173 studies in total of which 16 were ongoing and 157 were published which were included in this review for data extraction.

**Table 1: Summary of search results**

| PubMed | 273 |
| --- | --- |
| Ovid Embase | 549 |
| Scopus | 397 |
| Web of Science Core Collection | 693 |
| Cochrane CENTRAL | 47 |
| Global Index Medicus | 188 |
| Clinicaltrials.gov | 44 |
| WHO ICTRP | 80 |
| TOTAL | 2271 |
| Total after deduplication | 1336 |

**Reference**

1. Bush JT, Wasunna M, Alves F, Alvar J, Olliaro PL, Otieno M, et al. Systematic review of clinical trials assessing the therapeutic efficacy of visceral leishmaniasis treatments: A first step to assess the feasibility of establishing an individual patient data sharing platform. PLoS Negl. Trop. Dis. 2017;11:1–16.
