# Supplementary material for: Serious adverse events following treatment of visceral leishmaniasis: A systematic review and meta-analysis
Source: PLoS Negl Trop Dis. 2021 Mar 29;15(3):e0009302. doi: 10.1371/journal.pntd.0009302 (PMC8031744; doi:10.1371/journal.pntd.0009302)
Supplement: S3 Text — (DOCX) [file pntd.0009302.s003.docx]

# **S3 Text: Comparison of overall incidence rate of death between two groups**

Let $r_{1}$be the incidence rate of mortality for group 1 and $r_{2}$ be the rate for group 2. The interest is in estimating mean difference (D) in rate and **constructing 95% confidence interval** for *D*.

$$D=r_{1}-r_{2}$$

**Under the assumption of independence**, an expression for the variance of the rate difference can be obtained as:

$$Var\left( D \right)=var\left( r_{1} \right)+var\left( r_{2} \right)$$

The 95% confidence interval for rate difference is then:

$$D\pm1.96*standard error (D)$$

The default output of **metarate** command in R software returns the standard error of the rates on logarithm scale. The variance of rate can be approximated from the estimate of variance on the logarithm scale using delta rule. Following equation (2.8) in Collet (2015)^[[1]](#footnote-1)^, the variance of *g*(X) can be obtained using Taylor’s series approximation:

$$Var\left\{ g\left( X \right) \right\}\approx\left\{ \frac{d g\left( X \right)}{d X} \right\}^{2}Var(X)$$

$$\Rightarrow Var\left\{ log(X) \right\}\approx\left\{ \frac{d log(X)}{d X} \right\}^{2}Var(X)$$

$$\Rightarrow Var\left\{ log(X) \right\}\approx\left\{ \frac{1}{x} \right\}^{2}Var(X)$$

$$\Rightarrow Var\left\{ X \right\}\approx X^{2}.Var\left\{ log(X) \right\}$$

1. Collett D: *Modelling Survival Data in Medical Research, Third Edition*.*p26 (Equation 2.8)*. (2015) [↑](#footnote-ref-1)
