# Supplementary material for: Serious adverse events following treatment of visceral leishmaniasis: A systematic review and meta-analysis
Source: PLoS Negl Trop Dis. 2021 Mar 29;15(3):e0009302. doi: 10.1371/journal.pntd.0009302 (PMC8031744; doi:10.1371/journal.pntd.0009302)
Supplement: S1 Table — (DOCX) [file pntd.0009302.s004.docx]

# **S1 Table: Incidence rate of death in studies that allowed inclusion of patients with HIV co-infections**

|  | n/P/d | Random effects meta-analysis [95% CI] | I^2^ |
| --- | --- | --- | --- |
| **Drug regimen** |  |  |  |
| AmB-lipid | 2/1140/0 | - | - |
| L-AmB (multiple dose regimen) | 7/4020/2 | 0.469 [0.085–2.563] | 14.7% |
| L-AmB (multiple dose regimen) in a combination regimen | 1/1170/1 | 0.854 [0.120–6.067] | - |
| Miltefosine | 2/12300/7 | 0.569 [0.271–1.193] | 0.00% |
| Paromomycin | 1/6150/1 | 0.162 [0.022–1.154] | - |
| PA only | 6/27464/82 | 2.351 [0.754–7.327] | 95.0% |
| PA in a combination regimen | 2/3507/33 | 0.313 [0.223–0.441] | 0.0% |
| Overall | 21/5250/126 | 0.575 [0.244–1.355] | 92.0% |

n=number of study arms combined; d=total number of deaths within first 30 days of treatment initiation; P=Total person days; rates are expressed per 1,000 person-days; AmB=amphotericin B; AmB-lipid = Amphotericin b fat/lipid/colloid/cholesterol; L-AmB=Liposomal amphotericin B; PA=pentavalent antimony; CI=Confidence Interval; I^2^=measure of heterogeneity which quantifies the proportion of total variability that is due to between-study differences; the incidence rate of death (IRD) is estimated using a random effects Poisson regression
