# Supplementary material for: Serious adverse events following treatment of visceral leishmaniasis: A systematic review and meta-analysis
Source: PLoS Negl Trop Dis. 2021 Mar 29;15(3):e0009302. doi: 10.1371/journal.pntd.0009302 (PMC8031744; doi:10.1371/journal.pntd.0009302)
Supplement: S2 Table — (DOCX) [file pntd.0009302.s005.docx]

# **S2 Table: Sensitivity analysis assuming worst case scenario**

Worst case scenario estimates of incidence rate of death in first 30 days of treatment initiation in which all the cases of deaths with unclear time were considered to have occurred during the 30-day window

|  | (From Table 3 of main text)  Random effects estimates [95% CI] | | Worst case analysis^Ϯ^  Random effects estimates [95% CI] | |
| --- | --- | --- | --- | --- |
| **Drug name** | n/P/d | Random effects[95% CI] | n/P/d | Random effects [95% CI] |
| Sitamaquine | 17/296/1 | 0.112 [0.015–0.799] | 17/296/1 | 0.112 [0.015–0.799] |
| Pentamidine | 8/603/13 | 0.341 [0.072–1.605] | 8/603/13 | 0.341 [0.072–1.605] |
| Paromomycin | 14/1513/4 | 0.088 [0.033–0.234] | 14/1513/4 | 0.088 [0.033–0.234] |
| PA in a combination regimen | 20/4228/43 | 0.203 [0.054–0.762] | 20/4228/43 | 0.203 [0.054–0.762] |
| PA | 69/6596/158 | 0.215 [0.099–0.466] | 69/6596/173 | 0.227 [0.105–0.490] |
| Miltefosine | 31/4750/16 | 0.090 [0.036–0.225] | 31/4750/17 | 0.098 [0.041–0.234] |
| L-AmB (single dose) in a combination regimen | 8/945/2 | 0.070 [0.017–0.282] | 8/945/2 | 0.070 [0.017–0.282] |
| L-AmB (single dose) | 16/3271/2 | 0.017 [0.002–0.128] | 16/3271/2 | 0.017 [0.002–0.128] |
| L-AmB (multiple dose regimen) in a combination regimen | 4/408/2 | 0.147 [0.023–0.947] | 4/408/2 | 0.147 [0.023–0.947] |
| L-AmB (multiple dose regimen) | 49/1451/10 | 0.068 [0.010–0.435] | 49/1451/10 | 0.068 [0.010–0.435] |
| AMBd | 47/5250/32 | 0.069 [0.023–0.207] | 47/5250/33 | 0.065 [0.020–0.209] |
| AmB-lipid | 29/1267/2 | 0.052 [0.013–0.210] | 29/1267/2 | 0.052 [0.013–0.210] |
| **Overall** | 325/31706/285 | 0.068 [0.040–0.114] | 325/31706/302 | 0.069 [0.041–0.116] |

^Ϯ^Worst case scenario estimate of Incidence rate of death in first 30 days of treatment initiation where all the cases where deaths with unclear time were considered to have occurred during the 30-day window. All the cases where time of death were unclear were considered to have occurred during the first 30 days of treatment initiation

*n*=number of study arms combined; d=total number of deaths within first 30 days of treatment initiation; P=Total number of treated patients from all the arms which contributed to the meta-analysis; rates are expressed per 1,000 person-days; L- AmB=Liposomal amphotericin B; AmB-lipid = Amphotericin b fat/lipid/colloid/cholesterol; PA=pentavalent antimony; CI= Confidence Interval; the incidence rate of death (IRD) is estimated using a random effects Poisson regression
