# Supplementary material for: Serious adverse events following treatment of visceral leishmaniasis: A systematic review and meta-analysis
Source: PLoS Negl Trop Dis. 2021 Mar 29;15(3):e0009302. doi: 10.1371/journal.pntd.0009302 (PMC8031744; doi:10.1371/journal.pntd.0009302)
Supplement: S3 Table — (DOCX) [file pntd.0009302.s006.docx]

# **S3 Table: Mortality estimates stratified by randomisation status**

|  | Data from all study arms combined | | Only data from randomised studies combined | | Data from non-randomised/partially randomised or randomisation status not specified studies combined | |
| --- | --- | --- | --- | --- | --- | --- |
|  | n/P/d | RE [95% CI] | n/P/d | Random effects [95% CI] | n/P/d | Random effects [95% CI] |
| Sitamaquine | 17/296/1 | 0.112 [0.015–0.799] | 5/161/1 | 0.207 [0.029–1.469] | - | - |
| Pentamidine | 8/603/13 | 0.341 [0.072–1.605] | 4/189/1 | 0.176 [0.024–1.252] | 4/414/12 | 0.651 [0.135–3.134] |
| Paromomycin | 14/1513/4 | 0.088 [0.033–0.234] | 13/1393/4 | 0.095 [0.035–0.255] | - | - |
| PA combination regimen | 20/4228/43 | 0.204 [0.054–0.762] | 12/835/5 | 0.067 [0.005–0.914] | 8/3393/38 | 0.463 [0.224–0.954] |
| PA | 69/6596/158 | 0.215 [0.099–0.466] | 41/2615/44 | 0.051 [0.007–0.330] | 28/3981/114 | 0.621 [0.308–1.251] |
| Miltefosine | 31/4750/16 | 0.090 [0.036–0.225] | 10/504/6 | 0.208 [0.006–7.250] | 21/4246/10 | 0.078 [0.042–0.145] |
| L-AmB (single dose) in a combination regimen | 8/945/2 | 0.070 [0.017–0.282] | 6/452/1 | 0.073 [0.010–0.523] | 2/493/1 | 0.065 [0.006–0.659] |
| L-AmB (multiple dose regimen) in a combination regimen | 4/408/2 | 0.147 [0.023–0.947] | 4/408/2 | 0.147 [0.023–0.947] | - | - |
| L-AmB (multiple dose regimen) | 49/1451/10 | 0.068 [0.010–0.435] | 14/531/1 | 0.028 [0.000–18.565] | 35/920/9 | 0.129 [0.023–0.724] |
| AmBd | 47/5250/32 | 0.069 [0.023–0.207] | 31/3664/17 | 0.075 [0.021–0.269] | 16/1586/15 | 0.080 [0.011–0.544] |
| AmB-lipid | 29/1267/2 | 0.052 [0.013–0.210] | 16/1095/2 | 0.060 [0.015–0.243] | - | - |
| L-AmB (single dose) | 16/3271/2 | 0.017 [0.002–0.128] | - | - | 7/2667/2 | 0.021 [0.003–0.153] |
|  |  |  |  |  |  |  |
| Overall | 325/31706/285 | 0.068 [0.040–0.114] | 169/12922/84 | 0.046 [0.020–0.103] | 124/17007/185 | 0.129 [0.067–0.246] |

*n*=number of study arms combined; d=total number of deaths within first 30 days of treatment initiation; P=Total number of treated patients from all the arms which contributed to the meta-analysis; rates are expressed per 1,000 person-days; AmB-lipid = Amphotericin b fat/lipid/colloid/cholesterol; AmBd=Amphotericin B deoxycholate; L- AmB=Liposomal amphotericin B; PA=pentavalent antimony; CI= Confidence Interval; estimates are derived from random effects meta-analyses using Poisson regression
