# Supplementary material for: Serious adverse events following treatment of visceral leishmaniasis: A systematic review and meta-analysis
Source: PLoS Negl Trop Dis. 2021 Mar 29;15(3):e0009302. doi: 10.1371/journal.pntd.0009302 (PMC8031744; doi:10.1371/journal.pntd.0009302)
Supplement: S4 Table — (DOCX) [file pntd.0009302.s007.docx]

# **S4 Table: Assessment of risk of bias in randomised studies included in the review**

| **IDDO Tag** | **Study** | **Random sequence**  **generation** | **Allocation**  **concealment** | **Blinding of participants**  **and personnel** | **Blinding of**  **outcome assessment** | **Incomplete outcome**  **data addressed** | **Selective reporting** | **Adverse Events**  **Monitoring System**  **in Place** |
| --- | --- | --- | --- | --- | --- | --- | --- | --- |
| **1** | Ritmeijer-2006 | Low | Unclear | High | Unclear | High | Low | Low |
| **2** | Thakur-2001 | Unclear | Unclear | Unclear | Unclear | Low | Low | Low |
| **3** | Laguna-2003 | Low | Low | High | Low | Low | Low | Low |
| **5** | Sundar-2011a | Low | Low | High | Low | Low | Low | Low |
| **6** | Thakur-1994a | Unclear | Unclear | Unclear | Unclear | Low | Low | Low |
| **7** | Wali-1997 | Low | Unclear | Unclear | Unclear | Low | High | Low |
| **11** | Sundar-2008a | Low | Low | High | Low | Low | Low | Low |
| **12** | Sundar-2002a | Low | Unclear | High | Unclear | Low | Low | Low |
| **13** | Sundar-1999a | Low | Low | High | Low | Low | Low | Low |
| **14** | Sundar-2011b | Low | Low | High | Low | Low | Low | Low |
| **17** | Sundar-2000a | Low | Low | High | Low | Low | Low | Low |
| **18** | Laguna-1999 | Low | Unclear | High | Low | High | Low | Low |
| **22** | Chulay-1983 | Low | Unclear | High | High | Low | Low | Low |
| **23** | Das-2009 | Low | Unclear | High | Unclear | Low | Low | Low |
| **25** | Thakur-2000a | Low | Unclear | High | High | Low | Low | Low |
| **26** | Karki-1998 | Unclear | Unclear | High | Unclear | Low | Low | Low |
| **27** | Jha-2005 | Unclear | Unclear | High | Low | Low | Low | Low |
| **29** | Thakur-1995 | Unclear | Unclear | High | Unclear | High | High | Low |
| **30** | Sundar-2006 | Low | Low | High | Low | Low | Low | Low |
| **33** | Sundar-2004 | Low | Low | High | Low | Low | Low | Low |
| **34** | Sundar-2007a | Low | Low | High | Low | Low | Low | Low |
| **37** | Thakur-1996 | Unclear | Unclear | High | Unclear | Low | Low | Low |
| **38** | Thakur-1984a | Unclear | Unclear | Unclear | Unclear | Low | Low | Low |
| **39** | Thakur-1991a | Unclear | Unclear | Unclear | Unclear | Low | Low | Low |
| **40** | Thakur-2008 | Unclear | Unclear | Low | Unclear | Low | Low | Low |
| **43** | Thakur-1994c | Unclear | Unclear | Unclear | Unclear | Low | Low | Low |
| **51** | Thakur-1991b | Unclear | Unclear | Unclear | Low | Low | Low | Low |
| **54** | Hailu-2010 | Low | Low | High | Unclear | Low | Low | Low |
| **55** | Thakur-2010 | High | Low | High | Unclear | Low | Low | Low |
| **57** | Sundar-2007b | Low | Unclear | High | Unclear | Low | Low | Low |
| **61** | Sundar-2002b | Low | Low | Low | Low | Low | Low | Low |
| **63** | Musa-2010 | Low | Low | High | Unclear | Low | Low | Low |
| **69** | Jha-1998 | Low | Unclear | High | Low | Low | Low | Low |
| **70** | Thakur-1988 | Unclear | Unclear | Unclear | Low | Low | Low | Low |
| **72** | Khalil-2014 | Low | Low | High | Low | Low | Low | Low |
| **74** | Sundar-2009b | Low | Unclear | High | Unclear | Low | Low | Low |
| **76** | Sundar-1997 | Unclear | Low | High | Low | Low | Low | Low |
| **78** | Sundar-2010 | Low | Low | High | Low | Low | Low | Low |
| **79** | Musa-2012 | Low | Low | High | Unclear | High | Low | Low |
| **80** | Zijlstra-1993 | Unclear | Unclear | Unclear | Unclear | High | Low | Low |
| **81** | Sundar-1998a | Unclear | Low | High | Low | Low | Low | Low |
| **83** | Sundar-2001 | Low | Low | High | Low | Low | Low | Low |
| **86** | Thakur-2000b | Low | Unclear | High | Low | Low | Low | Low |
| **90** | Mondal-2010 | Low | Low | High | Unclear | Low | Low | Low |
| **92** | Mishra-1992 | Unclear | Unclear | High | Low | Low | Unclear | Low |
| **93** | Mishra-1994 | Unclear | Unclear | High | Unclear | Low | Low | Low |
| **95** | Thakur-1996 | Unclear | Unclear | High | Unclear | Low | Low | Low |
| **98** | Thakur-1993b | Unclear | Unclear | Unclear | Unclear | Low | Low | Low |
| **101** | Singh-2010 | Low | Unclear | Unclear | Unclear | High | Low | Low |
| **106** | Singh-2006b | Unclear | Unclear | Unclear | Unclear | Low | Low | Low |
| **108** | Karimi-1998 | Unclear | Unclear | Unclear | Unclear | Unclear | Unclear | Unclear |
| **119** | Thakur-1998b | Unclear | Unclear | Unclear | Unclear | Low | Low | Low |
| **120** | Das-2001 | Low | Unclear | Unclear | Unclear | Low | Low | Low |
| **121** | Sundar-199b | Low | Low | High | Low | Low | Low | Low |
| **133** | Sudarshan-2011 | Unclear | Unclear | Unclear | Unclear | Low | Low | Unclear |
| **138** | Thakur-1992b | Unclear | Unclear | Unclear | Unclear | Low | Unclear | Unclear |
| **140** | Goswami-2016 | Low | Low | High | Unclear | Low | Low | Low |
| **143** | Sundar-2014 | Low | Unclear | High | Unclear | High | Unclear | Low |
| **154** | Borges-2017 | Low | Low | High | Low | Low | Low | Low |
| **155** | Wasunna-2016 | Low | Low | High | Low | Low | Low | Low |
| **156** | Rahman-2017 | Low | Low | High | High | Low | Low | Low |
| **159** | Romero-2017 | Low | Low | High | Unclear | Low | Low | Low |
| **148** | Diro-2019 | Low | Low | High | Unclear | Low | Low | Low |
| **165** | Alborzi-2017 | Unclear | Unclear | High | Unclear | Low | Unclear | Low |
