# Supplementary material for: Serious adverse events following treatment of visceral leishmaniasis: A systematic review and meta-analysis
Source: PLoS Negl Trop Dis. 2021 Mar 29;15(3):e0009302. doi: 10.1371/journal.pntd.0009302 (PMC8031744; doi:10.1371/journal.pntd.0009302)
Supplement: S5 Table — (DOCX) [file pntd.0009302.s008.docx]

# **S5 Table: Incidence rate of death stratified by risk of bias status in randomised studies**

| Domain | Risk of bias  classification | n/P/d | Random effects  [95% confidence interval] | I^2^ |
| --- | --- | --- | --- | --- |
| Adverse events monitoring system in place | Low | 160/12761/84 | 0.047 [0.021–0.106] | 72.0% |
|  | Unclear | 9/161/0 | - | - |
| Allocation concealment | Low | 73/6601/33 | 0.100 [0.047–0.212] | 47.1% |
|  | Unclear | 96/6321/51 | 0.010 [0.001–0.102] | 86.4% |
| Blinding of participants and personnel | High | 119/9932/73 | 0.072 [0.035–0.147] | 64.0% |
|  | Low | 5/224/0 | - | - |
|  | Unclear | 45/2766/11 | 0.000 [0.000–1.975] | 92.6% |
| Incomplete outcome data addressed | High | 18/2897/47 | 0.197 [0.063–0.614] | 84.8% |
|  | Low | 148/9950/37 | 0.039 [0.014–0.105] | 60.7% |
|  | Unclear | 3/75/0 | - | - |
| Blinding of outcome assessment | High | 8/481/0 | - | - |
|  | Low | 74/5368/24 | 0.049 [0.014–0.169] | 62.8% |
|  | Unclear | 87/7073/60 | 0.054 [0.019–0.151] | 74.5% |
| Random sequence generation | High | 2/230/0 | - | - |
|  | Low | 95/9689/70 | 0.062 [0.028–0.139] | 69.9% |
|  | Unclear | 72/3003/14 | 0.014 [8e-04–0.252] | 80.1% |
| Selective reporting | High | 8/316/0 | - | - |
|  | Low | 147/11796/82 | 0.046 [0.019–0.111] | 74.0% |
|  | Unclear | 14/810/2 | 0.082 [0.020–0.329] | 0.0% |
| Overall for randomised studies | Overall | 169/12922/84 | 0.046 [0.020–0.103] | 71.5% |

n = number of study arms combined; d = total number of deaths within first 30 days of treatment initiation; P = Total person patients included from all the arms which contributed to the meta-analysis; rates are expressed per 1,000 person-days; CI = Confidence Interval; RE = Random effects meta-analysis; I^2^=measure of heterogeneity which quantifies the proportion of total variability that is due to between-study differences; the incidence rate of death (IRD) is estimated using a random effects Poisson regression
