# Supplementary material for: Serious adverse events following treatment of visceral leishmaniasis: A systematic review and meta-analysis
Source: PLoS Negl Trop Dis. 2021 Mar 29;15(3):e0009302. doi: 10.1371/journal.pntd.0009302 (PMC8031744; doi:10.1371/journal.pntd.0009302)
Supplement: S6 Table — (DOCX) [file pntd.0009302.s009.docx]

# **S6 Table: Assessment of risk of bias in studies other than randomised allocation of patients**

| **Study** | **Patient allocation** | **Bias due to  confounding ^a^** | **Bias in selection of participants into the study** | **Bias in classification  of interventions ^a^** | **Bias due to missing  outcome data** | **Selective reporting  of results** | **AEMS** |
| --- | --- | --- | --- | --- | --- | --- | --- |
| Giri-1993 | Single arm study | - | Moderate-serious | - | Unclear | Low | Low |
| Gaeta-2000 | Single arm study | - | Low | - | Low | Low | Low |
| Berhe-1999 | Single arm study | - | Low-moderate | - | Unclear | Unclear | Unclear |
| Jha-1999 | Alternating assignment | Moderate | Low | Low | Low | Low | Low |
| Sundar-2009a | Consecutive cohorts | Moderate-serious | Low | Low | Low | Low | Low |
| Sundar-2008a | Consecutive cohorts | Low-moderate | Low | Low | Low | Low | Low |
| Russo-1996 | Single arm study | - | Low-moderate | - | Low-moderate | Low | Low |
| Davidson-1994a | Single arm study | - | Low-moderate | - | Unclear | Unclear | Unclear |
| Thakur-2004a | Not specified | Low | Low | Low | Moderate | Low | Low |
| Wasunna-2005 | Consecutive cohorts | Moderate-serious | Low | Low | Low | Low | Low |
| Sundar-2011a | Single arm study | - | Low | - | Low | Low | Low |
| Thakur-1999 | Single arm study | - | Low | - | Low | Low | Low |
| Thakur-1993c | Single arm study | - | Serious | - | Low | Low | Low |
| Thakur-1994c | Not specified | Low | Serious | Low | Low | Low | Low |
| Moore-2001 | Alternating assignment | Low-moderate | Low | Low | Critical | Low | Low |
| Anabwani-1983 | Alternating assignment | Moderate-serious | Low | Low | Moderate | Low | Low |
| Sundar-1996 | Consecutive cohorts | Unclear | Low | Low | Low | Low | Low |
| Thakur-1998b | Single arm study | - | Low | - | Low | Low | Low |
| Bodhe-1999 | Not specified | Serious | Serious | Low | Unclear | Serious | Low |
| Freire-1997 | Not specified | Unclear | Serious | Low | Unclear | Low | Low |
| Sundar-2012 | Single arm study | - | Low | - | Low | Low | Low |
| Seaman-1993 | Alternating assignment | Low-moderate | Low | Low | Unclear | Unclear | Low |
| Thakur-2004b | Not specified | Critical | Low | Low | Unclear | Serious | Low |
| Ritmeijer-2001 | Alternating assignment | Low-moderate | Low | Moderate | Moderate | Low | Low |
| Jha-1983 | Single arm study | - | Serious | - | Unclear | Unclear | Low |
| Sundar-2000b | Single arm study | - | Low | - | Low | Low | Low |
| Rijal-2013 | Single arm study | - | Moderate | - | Low | Low | Low |
| Nyakundi-1994 | Not specified | Unclear | Low | Low | Unclear | Low | Low |
| Davidson-1994b | Not specified | Unclear | Low | Low | Low | Low | Low |
| Seaman-1995 | Consecutive cohorts | Moderate-serious | Low | Low | Critical | Low | Low |
| Thakur-2001b | Single arm study | - | Moderate-serious | - | Low | Low | Low |
| Sherwood-1994 | Consecutive cohorts | Low-moderate | Low | Low | Low | Low | Low |
| Dietze-2001 | Consecutive cohorts | Moderate-serious | Low | Low | Low | Low | Low |
| Rahman-2011 | Single arm study | - | Low | - | Low | Low | Low |
| Sinha-2011 | Single arm study | - | Low-moderate | - | Low | Low | Low |
| Bhattacharya-2007 | Single arm study | - | Low | - | Low-moderate | Low | Low |
| Mueller-2008 | Not specified | Serious | Low | Low | Low | Low | Low |
| Davidson-1996 | Consecutive cohorts | Low-moderate | Low | Low | Low | Low | Low |
| Sundar-2000a | Single arm study | - | Low-moderate | - | Low | Low | Low |
| Sundar-2003b | Single arm study | - | Low | - | Low | Low | Low |
| Dietze-1993 | Consecutive cohorts | Low-moderate | Low | Low | Low | Low | Low |
| Dietze-1995 | Single arm study | - | Low | - | Low | Low | Low |
| Chunge-1990 | Not specified | Unclear | Low | Low | Unclear | Low | Low |
| Thakur-1992b | Single arm study | - | Low | - | Low | Low | Low |
| Sundar-1998b | Consecutive cohorts | Unclear | Low | Low | Low-moderate | Low | Low |
| Jha-1995 | Single arm study | - | Moderate-serious | - | Low | Low | Low |
| Veeken-2000 | Alternating assignment | Moderate-serious | Low | Low | Low-moderate | Low | Low |
| Rijal-2010 | Single arm study | - | Low | - | Low | Low | Low |
| Berman-1998 | Partially Randomised | Serious-critical | Low | Low | Low | Low | Low |
| Patra-2012 | Single arm study | - | Low | - | Low | Low | Low |
| Mishra-1985 | Single arm study | - | Low | - | Low | Low | Low |
| Figueras Nadal-2003 | Single arm study | - | Low | - | Unclear | Low | Low |
| Castagnola-1996 | Consecutive cohorts | Unclear | Low | Low | Unclear | Unclear | Low |
| Thakur-1993a | Single arm study | - | Serious | - | Low | Low | Low |
| Bhattacharya-2004 | Single arm study | - | Low-moderate | - | Low | Low | Low |
| Singh-2006a | Single arm study | - | Low-moderate | - | Low | Low | Low |
| Sundar-2003a | Alternating assignment | Low-moderate | Moderate | Low | Low | Low | Low |
| di Martino-1997 | Alternating assignment | Low-moderate | Low | Low | Low-moderate | Low | Low |
| Syriopoulou-2003 | Single arm study | - | Moderate-serious | - | Low | Low | Low |
| Haidar-2001 | Single arm study | - | Low | - | Unclear | Unclear | Low |
| Tobaigy-1986 | Not specified | Unclear | Unclear | Low | Unclear | Unclear | Unclear |
| Sahay-1996 | Single arm study | - | Serious | - | Low | Low | Low |
| Mishra-1991 | Single arm study | - | Moderate-serious | - | Low | Low | Low |
| Das-2005 | Single arm study | - | Low | - | Unclear | Unclear | Low |
| Chowdhury-1991 | Single arm study | - | Serious-critical | - | Unclear | Unclear | Low |
| Rees-1984 | Single arm study | - | Low | - | Unclear | Low | Low |
| Rijal-2003 | Single arm study | - | Low | - | Low | Low | Low |
| Jha-1991 | Not specified | Low | Serious | Low | Unclear | Serious | Low |
| Singh-1995 | Not specified | Moderate-serious | Low | Low | Low | Low | Unclear |
| Giri-1994b | Single arm study | - | Moderate-serious | - | Low | Low | Low |
| Lal-1996 | Single arm study | - | Low | - | Unclear | Unclear | Low |
| Giri-1994a | Single arm study | - | Moderate-serious | - | Low | Low | Low |
| Thakur-1984a | Not specified | Critical | Serious | Low | Low | Low | Low |
| Jha-1998b | Single arm study | - | Low | - | Low | Unclear | Unclear |
| Ostyn-2014 | Single arm study | - | Low-moderate | - | Serious | Low | Low |
| Sinha-2010 | Single arm study | - | Low | - | Moderate | Low | Low |
| Mueller-2006 | Single arm study | - | Moderate-serious | - | Unclear | Unclear | Low |
| Cota-2014 | Not specified | Critical | Serious | Low | Low | Low | Low |
| Sundar-1998a | Consecutive cohorts | Low-moderate | Low | Low | Unclear | Unclear | Low |
| Adam-2009 | Single arm study | - | Low | - | Unclear | Moderate | Low |
| Shahian-2009 | Single arm study | - | Moderate | - | Unclear | Low | Low |
| Villanueva-2000 | Not specified | Unclear | Low | Low | Serious-critical | Low | Low |
| Thakur-1998a | Single arm study | - | Moderate-serious | - | Unclear | Unclear | Low |
| Jamil-2015 | Single arm study | - | Low | - | Low | Low | Low |
| Sundar-2015 | Consecutive cohorts | Low-moderate | Low | Low | Low | Low | Low |
| Mondal-2014 | Single arm study | - | Low-moderate | - | Low | Low | Low |
| Rashid-1994 | Single arm study | - | Low | - | Unclear | Unclear | Low |
| Sundar-2019 | Single arm study | - | Low | - | Unclear | Low | Low |
| Mbui-2018 | Single arm study | - | Low | - | Low | Low | Low |
| Pandey-2017 | Single arm study | - | Low | - | Low | Low | Low |
| Pandey-2016 | Single arm study | - | Low | - | Low-moderate | Low | Low |
| Kimutai-2017 | Single arm study | - | Moderate-serious | - | Low | Low | Low |
| Goyal-2018 | Not specified | Critical | Low | Low | Low | Low | Low |

AEMS= Adverse Events Monitoring System; ^a^ These domains are not assessed for single arm studies
