# Supplementary material for: Serious adverse events following treatment of visceral leishmaniasis: A systematic review and meta-analysis
Source: PLoS Negl Trop Dis. 2021 Mar 29;15(3):e0009302. doi: 10.1371/journal.pntd.0009302 (PMC8031744; doi:10.1371/journal.pntd.0009302)
Supplement: S7 Table — (DOCX) [file pntd.0009302.s010.docx]

# **S7 Table: Incidence rate of death stratified by risk of bias status in studies other than randomised allocation of patients**

| Domain | Risk of bias | n/P/d | Random effects  [95% confidence interval] | I^2^ |
| --- | --- | --- | --- | --- |
| Bias due to confounding | Low | 7/3606/41 | 0.359 [0.106–1.214] | 77.7 |
|  | Moderate | 32/993/63 | 0.135 [0.016–1.098] | 89.9 |
|  | High | 41/4117/50 | 0.071 [0.012–0.410] | 90.0 |
|  | Unclear | 24/278/2 | 0.239 [0.060–0.958] | 0.0 |
|  | Single arm studies | 52/9790/45 | 0.119 [0.060–0.235] | 68.5 |
| Bias in participant selection | Low | 111/10176/142 | 0.140 [0.066–0.298] | 85.1 |
|  | Moderate | 13/1786/4 | 0.079 [0.021–0.292] | 36.1 |
|  | High | 30/6806/55 | 0.109 [0.033–0.366] | 79.9 |
|  | Unclear | - | - | - |
| bias in interventions classification | Low | 102/8795/108 | 0.116 [0.042–0.318] | 82.7 |
|  | Moderate | 2/199/48 | 8.038 [6.015–10.742] | 0.6 |
|  | High | - | - | - |
|  | Unclear | - | - | - |
|  | Single arm studies | 52/9790/45 | 0.119 [0.060–0.235] | 68.5 |
| Missing outcome data | Low | 88/11235/81 | 0.083 [0.036–0.194] | 74.4 |
|  | Moderate | 21/2418/77 | 0.365 [0.104–1.280] | 91.0 |
|  | High | 6/1167/10 | 0.776 [0.137–4.381] | 85.8 |
|  | Unclear | 41/3964/33 | 0.135 [0.040–0.456] | 77.4 |
| bias in outcome assessment | Low | 156/18784/201 | 0.120 [0.065–0.222] | 84.3 |
|  | Moderate | - | - | - |
|  | High | - | - | - |
|  | Unclear | - | - | - |
| Selective Reporting | Low | 128/16188/169 | 0.099 [0.048–0.204] | 85.7 |
|  | Moderate | 1/42/4 | 3.174 [1.191–8.458] | - |
|  | High | 8/462/8 | 0.273 [0.039–1.905] | 68.5 |
|  | Unclear | 19/2092/20 | 0.220 [0.062–0.780] | 66.4 |
| AEMS in Place | Low | 147/18587/201 | 0.129 [0.070–0.236] | 84.7 |
|  | Moderate | - | - | - |
|  | High | - | - | - |
|  | Unclear | 9 | - | - |

n=number of study arms combined; d=total number of deaths within first 30 days of treatment initiation; P=Total person patients included from all the arms which contributed to the meta-analysis; rates are expressed per 1,000 person-days; CI=Confidence Interval; RE=Random effects meta-analysis; AEMS = adverse events monitoring system; I^2^=measure of heterogeneity which quantifies the proportion of total variability that is due to between-study differences; the incidence rate of death (IRD) is estimated using a random effects Poisson regression
